# Description, Staging and Quantification of Pulmonary Artery Angiophagy in a Large Animal Model of Chronic Thromboembolic Pulmonary Hypertension

**DOI:** 10.3390/biomedicines8110493

**Published:** 2020-11-11

**Authors:** Frédéric Perros, Maria-Rosa Ghigna, Fanny Loisel, Denis Chemla, Benoit Decante, Vincent de Montpreville, David Montani, Marc Humbert, Elie Fadel, Olaf Mercier, David Boulate

**Affiliations:** 1 School of Medicine, Université Paris-Saclay, 94270 Le Kremlin-Bicêtre, France; frederic.perros@inserm.fr (F.P.); mr.ghigna@hml.fr (M.-R.G.); denis.chemla@aphp.fr (D.C.); david.montani@aphp.fr (D.M.); marc.humbert@aphp.fr (M.H.); e.fadel@ghpsj.fr (E.F.); o.mercier@hml.fr (O.M.); 2 INSERM UMR_S 999 «Pulmonary Hypertension: Pathophysiology and Novel Therapies», Hôpital Marie Lannelongue, 92350 Le Plessis-Robinson, France; 3 Research and Innovation Unit, Hôpital Marie Lannelongue, 92350 Le Plessis-Robinson, France; fanny.loisel@outlook.fr (F.L.); b.decante@ghpsj.fr (B.D.); 4 Department of Pathology, Hôpital Marie Lannelongue, 92350 Le Plessis-Robinson, France; v.thomasdemontpreville@ghpsj.fr; 5 Department of Physiology, Hôpital Bicêtre, Université Paris-Saclay, 94270 Le Kremlin-Bicêtre, France; 6 Assistance Publique-Hôpitaux de Paris (AP-HP), Department of Respiratory and Intensive Care Medicine, Pulmonary Hypertension National Referral Center, Hôpital Bicêtre, 94270 Le Kremlin-Bicêtre, France; 7 Department of Thoracic and Vascular Surgery and Heart-Lung Transplantation, Marie Lannelongue Hospital, 92350 Le Plessis Robinson, France

**Keywords:** pulmonary embolism, angiophagy, pulmonary artery, remodeling, pathology, animal model, pulmonary hypertension

## Abstract

Angiophagy has been described as a non-fibrinolytic mechanism of pulmonary artery (PA) patency restoration after distal (<50 µm in diameter) pulmonary embolism in mice. We hypothesized that angiophagy could achieve muscularized PA patency restoration after pulmonary embolism in piglets and humans. Angiophagy was defined by pathological assessment as the moving of an embolic specimen from the lumen to the interstitium according to three stages in a pig model of chronic thromboembolic pulmonary hypertension (CTEPH) 6 to 10 weeks after embolization with enbucrilate: the embolic specimen is (I) covered by endothelial cells, (II) covered by endothelial cells and smooth muscle cells, and (III) located in the adventitia. In animals, we observed the three stages of the pulmonary angiophagy of enbucrilate emboli in <300 µm PA. Stages II and III were observed in 300 to 1000 μm PA, and only Stage I was observed in larger-diameter PA (>1000 μm). In lung samples from patients with histories of pulmonary embolisms, we observed PA angiophagy stigma for embolic specimens derived from blood clots and from bone marrow emboli. This study provides an original pathological description and staging of PA angiophagy in a large animal model of CTEPH and in humans after pulmonary embolism.

## 1. Introduction

Over a lifetime, hundreds of millions of liters of blood will go through the pulmonary arteries. Pulmonary artery physiology allows for high flow–low pressure–low resistance circulation between the right ventricle and the pulmonary veins. The obstruction of the pulmonary arteries after a pulmonary embolism can lead to hemodynamic compromise and death, such as in acute pulmonary embolism or chronic thromboembolic pulmonary hypertension (CTEPH). After an acute pulmonary embolism, it is well established that in survivors, pulmonary circulation patency tends to be restored within a few weeks [1]. Embolic specimens are mostly constituted of blood clots; nevertheless, a variety of constituents may generate emboli such as bone marrow fragments, amniotic liquid or even exogenous particles accidentally introduced within the circulation [2,3]. The clearance of a thromboembolic specimen after an acute pulmonary embolism is the result of the fibrinolytic system and hemodynamic forces, leading to embolus decay and washout [4,5]. However, the fibrinolytic system is limited to breaking down fibrin-based blood clots and is unable to disrupt other materials, such as calcium and cholesterol crystals and cell debris, which are present in complex thrombi [6]. Two observations reported in the literature suggest that there may exist at least one additional non-fibrinolytic mechanism leading to pulmonary artery clearance in cases of embolic specimens in the pulmonary artery lumen. First, it is difficult to achieve chronic pulmonary artery obstruction and pulmonary hypertension in animal models of iterative pulmonary embolism even when non-thrombotic embolic specimens such as ceramic beads or biological glue are used [4,7,8]. Second, Tomashefski et al. described embolized foreign bodies inside and also outside the pulmonary arteries in the lungs of patients who used illicit intravenous drug injections [2,3].

Angiophagy is defined as the ability of the vessel wall to envelop emboli and to remove them from micro-vessels [9]. This non-fibrinolytic mechanism involved in the preservation of very distal (<50 µm diameter) vascular patency was described in the systemic and pulmonary microcirculations of mice to remove blood clots and polystyrene microspheres. Pulmonary artery angiophagy was described in mice, in relatively small pulmonary arteries (<50 µm in diameter). However, to our knowledge, pulmonary artery angiophagy has not been described in large animals and humans or in muscularized pulmonary arteries. As it may have major impacts on our understanding of pulmonary artery physiology and pathophysiology, we chose to perform a systematic analysis of the pulmonary arteries of our large animal model of chronic thromboembolic pulmonary hypertension (CTEPH), in which we repeatedly embolized a biological glue (enbucrilate) in the right lower lobe pulmonary arteries once a week for 5 weeks [10,11,12], and of lung specimens from patients who underwent lung transplantations for chronic thromboembolic diseases. We hypothesized that pulmonary angiophagy may be observed in pulmonary arteries within weeks after iterative pulmonary embolisms of different natures, and that it could be described by different pathological stages as a physiological process.

We first performed a morphometric analysis of the process of angiophagy in the right lower lobes of our large animal model of CTEPH. We identified three consecutive stages in the process of angiophagy from the transition of the embolic specimen under the endothelial cell layer to its location outside of the pulmonary artery adventitia. Then, we described the process in specimens of human lungs including different pulmonary artery diameters and for various types of embolic specimen in patients who underwent lung transplantations for pulmonary thromboembolic diseases. Finally, we discuss the role of this mechanism in chronic vascular remodeling.

## 2. Methods

This study complied with the principles of laboratory animal care according to the National Society for Medical Research and was approved by the local ethics committee for animal experiments at Marie Lannelongue Hospital. Regarding human data, ethical approval was obtained for all the experiments according to current national and international laws (Comité de Protection des Personnes EST-III, N° CPP: 18.06.06; Authorization, N°ID RCB: 2018-A01252-53, 19 July 2018).

### 2.1. Animal Model

**Figure 1:**
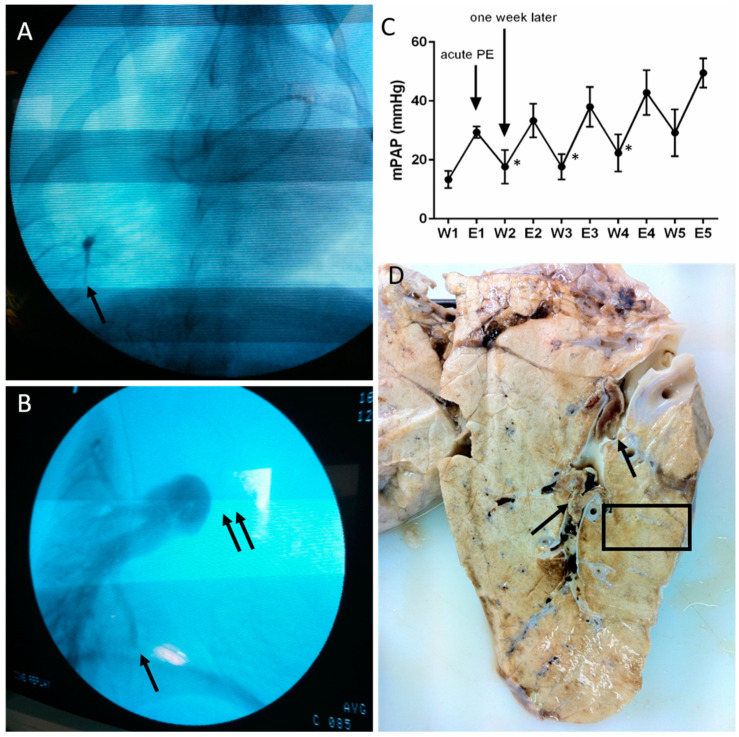
Imaging, hemodynamics and gross anatomy associated with iterative embolism of the right lower lobe in our large animal model of chronic thromboembolic pulmonary hypertension. (**A**) Thoracic fluoroscopy after an acute pulmonary embolism of the right lower lobe. The arrows show the embolic specimen of right lower lobe pulmonary arteries with enbucrilate mixed with lipiodol (contrast dye). (**B**) Pulmonary arteriography showing complete occlusion of the left pulmonary artery (double arrow) and distal pulmonary artery occlusion in the right lower lobe (simple arrow). (**C**) Iterative measurements of mean pulmonary artery pressure (mean ± standard deviation) before (Wn) acute pulmonary embolism and immediately after (En), *n* being the week of acute pulmonary embolism. * *p* < 0.05 compared to the previous value. (**D**) Gross anatomy of right lower lobe; the arrows show chronic proximal pulmonary artery occlusions; the block rectangle shows the limits of the pathological specimens used for histopathological analysis.

We induced a CTEPH model as previously described [10,12] in 11 “large white” piglets aged 6 weeks old, at the initiation of the protocol, weighing (mean ± standard deviation) 23.2 ± 5.4 kg. To achieve CTEPH modeling, we performed a ligation of the left pulmonary artery and iterative embolization of the right lower lobe, once a week for 5 weeks, utilizing a biological glue (enbucrilate, Histoacryl^®^, B.Braun, Melsungen, Germany) mixed with an equivalent volume of lipiodol (Figure 1A). Each week, we injected 1 to 4 mL of the enbucrilate–lipiodol solution into the right lower pulmonary artery through a venous catheter inserted percutaneously through the jugular vein under fluoroscopic control. This induced a progressive pulmonary artery occlusion (Figure 1B). Pulmonary artery pressures were measured using a Swan–Ganz catheter as previously described [12]. The mean pulmonary artery pressure (mPAP) after acute pulmonary artery embolization at Weeks 1, 2 and 3 (E1, E2 and E3, respectively) significantly decreased after one week at W2, W3 and W4, respectively (all *p* < 0.05; Figure 1C). Eleven animals were sacrificed at 6 weeks after the last pulmonary artery embolization (11 weeks after the first embolization), and the right lower lobe was harvested (Figure 1D).

### 2.2. Histopathological Analysis of Pulmonary Artery Angiophagy in Right Lower Lobe of the CTEPH Model

The right lower lobes of 11 animals were harvested and fixed in 10% buffered formalin for 24 h. Random samples were embedded in paraffin and cut at a 5 µm thickness. Lung tissues were stained with hematoxylin and eosin and examined under light microscopy. Two to three samples were analyzed per animal, corresponding to 32 samples of right lower lobes with a mean (±standard deviation) of 2.67 ± 0.49 samples per animal. Morphometric measurements were performed using imaging software (NIS-Element BR, version 2.30; Nikon USA, Melville, NY, USA).

**Figure 2:**
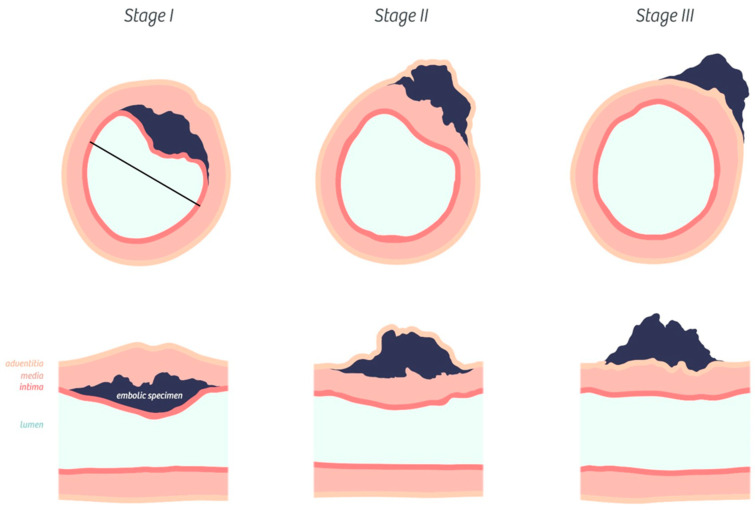
Classification of pulmonary artery angiophagy. Embolic specimen progression through the pulmonary arterial wall is shown in axial cuts (upper row) and in a longitudinal cut (lower row). The black line represents the estimated shortest pulmonary artery diameter measurement in case of concentric remodeling.

For each angiophagic lesion, we report its stage and the inner diameter of the concerned pulmonary artery. The inner diameter was measured by tracing the pulmonary artery (PA) diameter corresponding to the observed or supposed shortest diameter between opposing sub-endothelial (internal border of the media) layers. Indeed, eccentric or concentric remodeling required the measurement of the supposed shortest diameter to minimize measurement errors because this remodeling could artificially reduce the pulmonary artery diameter (Figure 2). As there is no existing classification of angiophagy stages, we described the angiophagic process in three stages as follows—Stage I: the embolic specimen is covered by endothelial cells; Stage II: the embolic specimen is covered by endothelial cells and smooth muscle cells; and Stage III: the embolic specimen is located in adventitia (Figure 2). The location of the embolic specimen and stages were reported by a pathologist (M.-R.G.).

### 2.3. Human Lung Sample Analysis

Based on the animal model results and angiophagy classification, we analyzed available samples of human lung tissues from two patients transplanted for end-stage pulmonary hypertension with histories of pulmonary embolism. We report the type of embolic specimen and the angiophagic lesion stage.

### 2.4. Immunohistochemistry

Tissue sections were deparaffinized in xylene and rehydrated through decreasing concentrations of alcohol. The immunostainings, using CD31 (QBEnd/10 mouse monoclonal) and smooth muscle actin antibodies, were performed according to the facility’s automated protocol (Benchmark GX Autostainer, Ventana Medical System, Roche, Bâle, Switzerland). Vessels’ elastic lamina were highlighted by fuchsin–resorcin staining.

### 2.5. Statistical Analysis

Continuous variables are reported as mean ± standard deviation. Paired values were compared using non-parametric Wilcoxon matched-pair signed rank tests, and unpaired values, using Mann–Whitney tests. The relationships between categorical variables were evaluated using *Chi*^2^ tests. The statistical analyses were performed using *GraphPad Prism 8.3.0* (GraphPad Software, LLC). *p* < 0.05 was considered significant.

## 3. Results

### 3.1. Histopathological Assessment of Angiophagy in Right Lower Lobes of the CTEPH Animal Model

**Figure 3:**
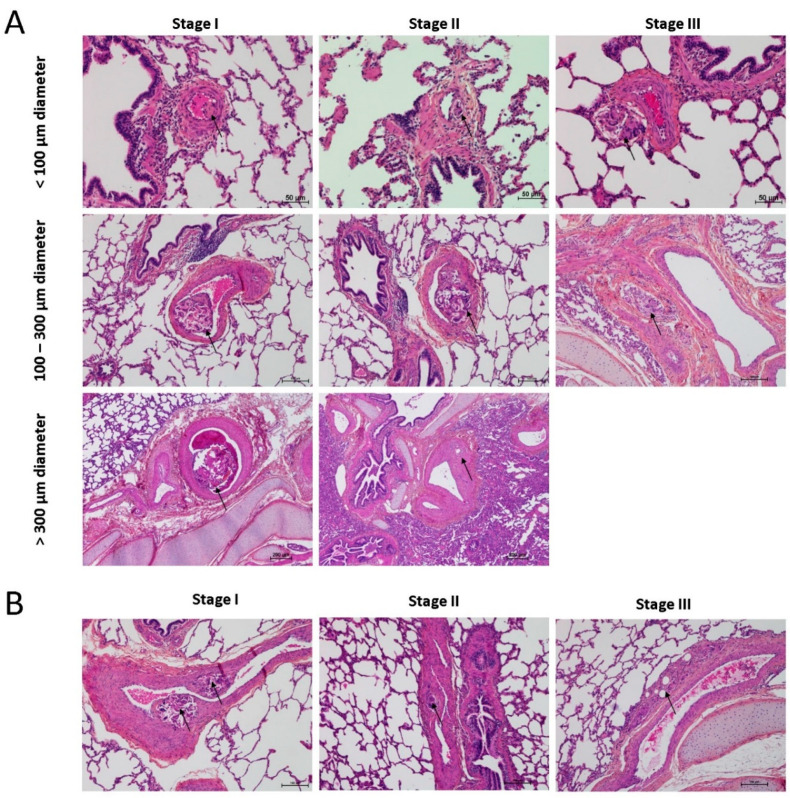
Histopathology of right lower lobes of the animal model of chronic thromboembolic pulmonary hypertension (CTEPH) showing axial (**A**) and longitudinal (**B**) arterial cuts of the 3 stages of angiophagy (3 rows). Arrows show embolic specimen position. Stain is hematein–eosin–saffron.

**Figure 4:**
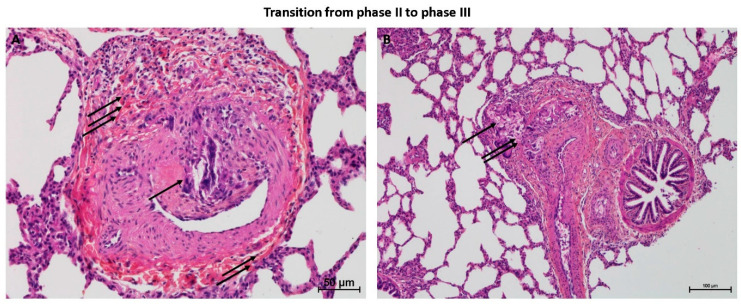
Histopathology of transition between phase II and phase III pulmonary angiophagy in the large animal model of CTEPH (axial cut). (**A**) The embolic specimen (simple arrow) is engulfed into the pulmonary artery wall and covered by endothelial and smooth muscle cells. The double arrow shows a structured collagen layer on the opposite side of the angiophagy process. The triple arrows show a destructured collagen layer on the side of the angiophagy. (**B**) The embolic specimen (single arrow) is situated on both sides of the collagen layer, which shows discontinuity (double arrow). Stain is hematein–eosin–saffron.

**Figure 5:**
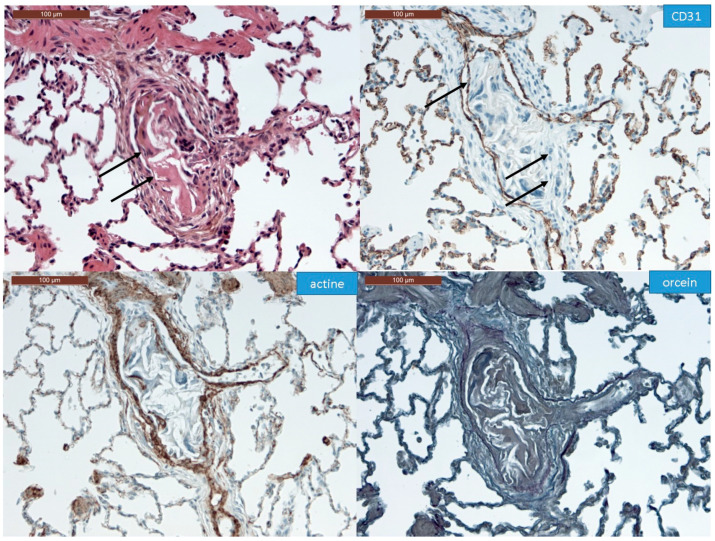
Immunohistochemistry of Stage I angiophagy in a large animal model of CTEPH (axial cut). The hematein–eosin–saffron stain (left upper panel) shows an intraluminal thrombus (arrows). The intraluminal embolic specimen is covered by endothelial cells highlighted with CD31 staining (arrow). There is a disappearance of endothelial cells on the arterial wall side of the embolic specimen (double arrow, right upper panel). There is neither disruption of the smooth muscle cell layer of the media as shown by the actin staining, nor of the elastic lamina stained with fuchsin–resorcin (orcein).

**Figure 6:**
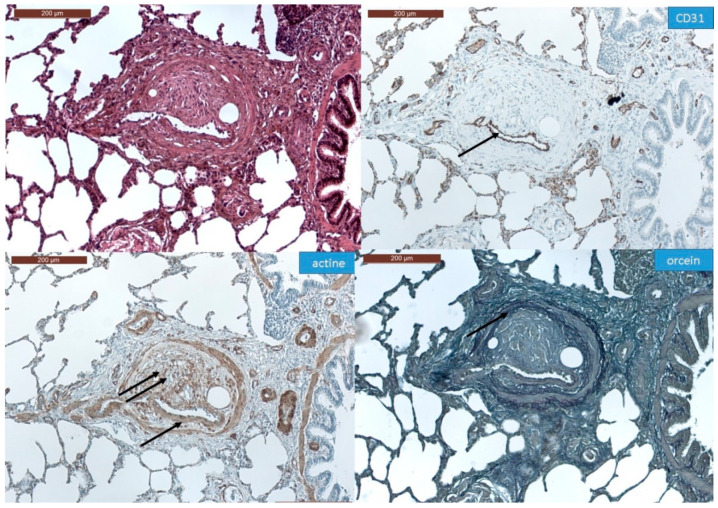
Immunohistochemistry of Stage II angiophagy in the large animal model of CTEPH (axial cut). The hematein–eosin–saffron coloration (left upper panel) shows a cellularized embolic specimen in the pulmonary artery wall. The embolic specimen is covered by endothelial cells (arrow, CD31 staining, right upper panel). The smooth muscle cell layer of the media is disrupted at the contact of the embolic specimen (double arrow, actin staining, left lower panel), whereas it remains intact on the opposite side of the pulmonary artery (single arrow, left lower panel). The fuchsin–resorcin (orcein) staining (right lower panel) shows an intact elastic lamina (arrow).

**Figure 7:**
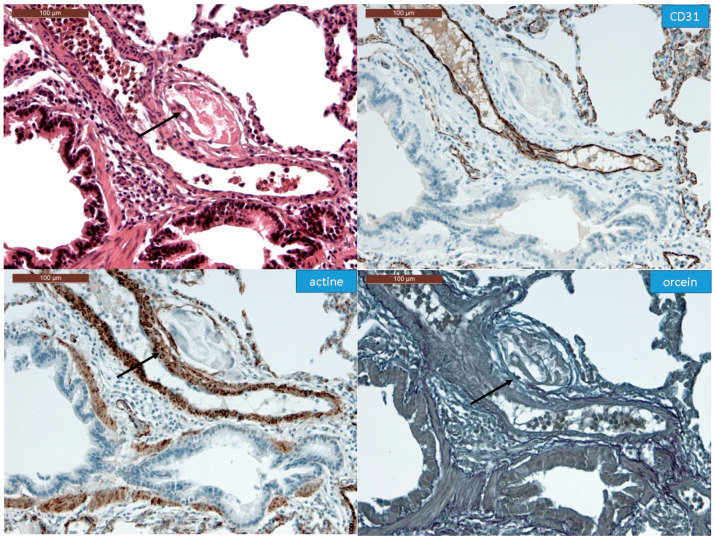
Immunohistochemistry of Stage III angiophagy in the large animal model of CTEPH (longitudinal cut). The hematein–eosin–saffron coloration (left upper panel) shows an embolic specimen (arrow) outside the media in the adventitia of the pulmonary artery. The embolic specimen is separated from the pulmonary artery lumen by endothelial cells (CD31 staining, right upper panel, arrow) and organized smooth muscle cells (actin staining, left lower panel, arrow). The adventitia seems disrupted at the contact of the embolic specimen (orcein staining, right lower panel, arrow).

In all the animal tissues, the embolic vascular obstruction appeared as “foreign body” granulomas containing white droplets of enbucrilate. The angiophagy pattern was observed for three stages (Figure 2, Figure 3 and Figure S1). We also observed some images illustrating the transition between Stages II and III (Figure 4). The immunohistopathological staining of endothelial cells, smooth muscle cells and elastic lamina further illustrated the three stages. Stage I was characterized by an intimal adhesion of the embolic specimen enveloped by endothelial cells with the disappearance of endothelial cells on the arterial wall side of the embolic specimen and the beginning of the disruption of the internal elastic lamina (Figure 5). Stage II was characterized by a transvascular migration of the embolic agent. This stage is marked by a profound destructuring of the vessel wall, with a disruption of the elastic lamina and vascular smooth muscle cell spacing, opening a “path” for the embolus to move through (Figure 6). Stage III was characterized by embolic specimen extrusion and storage within the perivascular connective tissue (Figure 7).

### 3.2. Quantitative Morphological Analysis of Angiophagy in Right Lower Lobes of the CTEPH Animal Model

**Figure 8:**
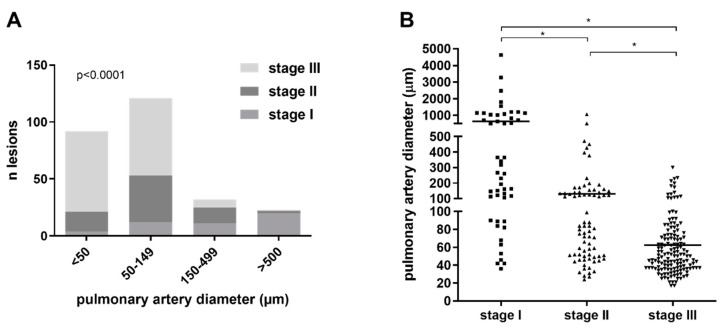
**Quantification of angiophagy in right lower lobes of the large animal model of CTEPH.** (**A**) Number (*n*) of each stage of angiophagic lesions among four different diameters of pulmonary arteries (axial cut); proportions of stages among diameter ranges were compared using *Chi*^2^ tests. (**B**) Distribution of pulmonary artery diameter for each stage of pulmonary angiophagy (axial cut); the bar represents the median value; pulmonary artery diameters corresponded to the inner diameters measured between the inner borders of the media and were compared using Mann–Whitney tests; * *p* < 0.05.

We observed 274 angiophagic lesions in pulmonary arteries (PA) with an internal diameter of 17 to 4630 μm. In the smallest PA (<150 μm diameter), there was a majority of Stage III angiophagic lesions, whereas in the larger PA (≥150 μm diameter), there was a majority of Stage I and II angiophagic lesions (Figure 8A). There was a significant difference in the proportions of pulmonary artery angiophagy stages (I, II or III) among the categories of pulmonary artery diameters (<50, 50–149, 150–499 or >500 µm; *Chi*^2^ test, *p* < 0.0001; Figure 8A). We observed 49 Stage I angiophagic lesions in <5000 µm-diameter pulmonary arteries, 76 Stage II angiophagic lesions in <1000 µm-diameter pulmonary arteries, and 149 Stage III angiophagic lesions in <300 µm-diameter pulmonary arteries (Figure 8B). The mean (±standard deviation) diameter of the pulmonary arteries in which Stage I lesions were observed was significantly larger than the diameter of the pulmonary arteries in which Stage II and III were observed (Stage I: 642.8 ± 886.2 µm, Stage II: 129.9 ± 152.2 and Stage III: 62.43 ± 43.20, all *p* < 0.05, Figure 8B). Stage I, II and III angiophagic lesions were observed in nine animals; in one animal, only Stages II and III were observed; in another animal, only Stages I and II were observed.

### 3.3. Histopathology of Pulmonary Angiophagy in Human Samples

**Figure 9:**
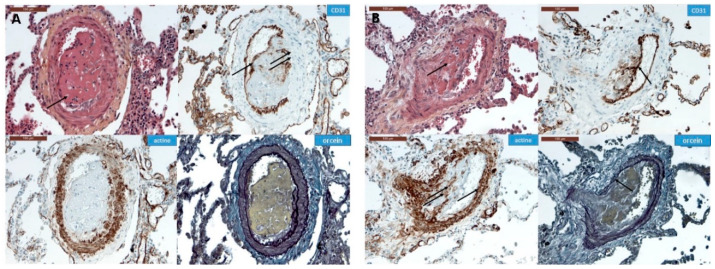
Immunohistochemistry of Stage I and Stage II angiophagy in humans: a patient with CTEPH (axial cut). (**A**) (Stage I): Hematein–eosin–saffron (HES) staining shows an intraluminal thrombus (arrow, left upper panel). CD31 staining of the endothelial cells (right upper panel) shows that the thrombus is separated from the lumen by a layer of endothelial cells (single arrow); meanwhile, the thrombus is in direct contact with the smooth muscle cells (double arrow). The smooth muscle cell layer stained with anti-actin antibodies (left lower panel) and the elastic lamina stained with fuchsin–resorcin (orcein, right lower panel) remain intact. (**B**) (Stage II): Hematein–eosin–saffron coloration (left upper panel) shows a thrombus in the pulmonary artery wall (arrow). The thrombus is covered by endothelial cells (arrow, CD31 staining, right upper panel). The smooth muscle cell layer of the media is disrupted at the contact with the thrombus (double arrow, actin staining, left lower panel), whereas it remains intact at the opposite side of the pulmonary artery (single arrow, left lower panel). The fuchsin–resorcin staining (right lower panel) shows an intact elastic lamina at contact with the thrombus (arrow).

**Figure 10:**
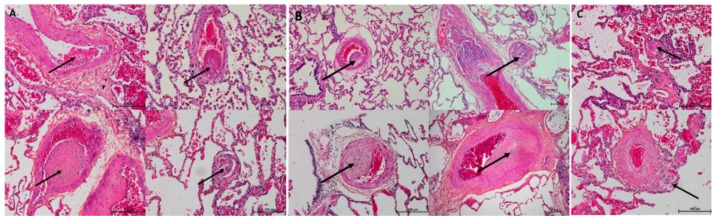
Histopathology of different patterns of thrombi for the 3 stages of angiophagy in patients with CTEPH. (**A**) (Stage I): In the left upper panel, a recent thrombus of fibrin is poorly cellularized; in the right upper panel, the thrombus seems adherent to the media and is surrounded by endothelial cells with few cells inside the thrombus; in the left lower panel, the thrombus is, importantly, cellularized with a low rate of fresh fibrin, which reflects an aspect of fibrinous evolution; in the right lower panel, the thrombus seems entirely cellularized without fibrin or fibrosis. (**B**) (Stage II): The different patterns of thrombi were cellularized thrombi without fibrosis (left and right upper panels) and cellularized thrombi with fibrosis (left and right lower panels). (**C**) (Stage III). The thrombus is located outside the pulmonary artery (arrow) and is poorly cellularized (upper panel); the thrombus is located outside the pulmonary artery (arrow) and is cellularized (lower panel). Hematein–eosin–saffron staining.

**Figure 11:**
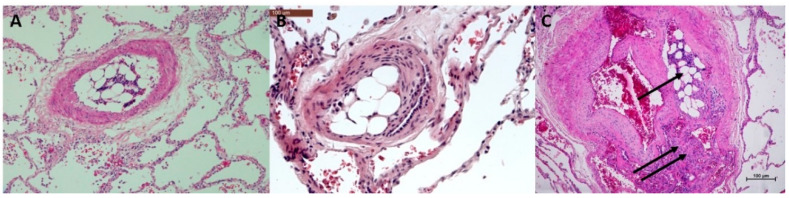
Histopathology of non-fibrinous embolic specimen in humans. (**A**) Occlusive embolic specimen of bone marrow without angiophagy. (**B**) Stage II angiophagy of a bone marrow embolic specimen. (**C**) Stage II angiophagy of a bone marrow embolic specimen (single arrow) and of a cellularized fibrinous thrombus (double arrow). Stain is hematein–eosin–saffron.

To make a proof of concept showing that angiophagy as described by J. Grutzendler et al. [6] in mice exists in humans, we took advantage of the availability in our center of samples of human lung tissues from patients transplanted with histories of pulmonary embolisms. Human samples were retrieved from the pathology department archive. Both were samples of native lungs of two patients with chronic thromboembolic diseases who had undergone lung transplantation. One patient was a 22-year-old female, clinically presenting with progressive dyspnea and cardiac impairment. Preoperative hemodynamic evaluation confirmed pulmonary hypertension (systolic pulmonary artery pressure: 88 mmHg). This patient underwent a heart–lung transplantation as a salvage after a failed pulmonary endarterectomy due to very distal thromboembolic disease not accessible for pulmonary endarterectomy. The samples used for histological evaluation were retrieved from the right lung (two blocks from the right upper lobe and three blocks from the right lower lobe). The second patient was a 58-year-old male with Klippel–Trenaunay disease, presenting with severe dyspnea. A preoperative hemodynamic evaluation confirmed pulmonary hypertension (systolic pulmonary artery pressure: 79 mmHg). This patient underwent a double lung transplantation for end-stage right heart failure secondary to chronic thromboembolic pulmonary hypertension with distal disease. The lung histology was assessed on two samples from the upper right lobe and three samples from the lower right lobe. In these samples, we observed the three stages of pulmonary angiophagy in each patient. In the first patient, we found five type I lesions, six type II lesions and three type III lesions; in the second patient, we found 18 type I lesions, 20 type II lesions and four type III lesions. Similar to in the animal model, the embolic specimens (fibrin thrombi) were enveloped by endothelial cells and also with spindle cells expressing smooth muscle actin. Immunohistochemistry showed that the fibrin thrombi can become increasingly cellular before engaging through the vessel wall from Stage I (Figure 9A) to Stage II (Figure 9B). However, we observed differently cellularized types of fibrin thrombi for each stage of angiophagy (Figure 10 and Figure S2). Stage III angiophagy for fibrin thrombi was observed in small arteries (<70 µm in diameter), but this rarely compared with the animals. Persistent arterial remodeling was characterized by an eccentric and concentric fibrous increase inf the intima with a narrowing or disappearing lumen. This remodeling was observed in most pulmonary arteries ranging from 100 to 500 µm. We observed at least two types of embolic specimen: fibrin thrombi derived from blood clots and bone marrow embolic specimens (Figure 11).

## 4. Discussion

To the best of our knowledge, this is the first study showing that the pulmonary artery wall may be able to move pulmonary artery embolic specimens from the lumen to the interstitium, in humans and in piglets. This process, which has only been described recently in the mouse microcirculation, was termed “angiophagy”. Therefore, our study is also the first description of angiophagy in muscularized pulmonary arteries. Consequently, angiophagy could play a pivotal role in maintaining micro-vessels and muscularized vessel patency by removing blood clots, cell debris, bone marrow or foreign bodies from the pulmonary artery lumen. The first descriptions of angiophagy illustrated its effectiveness in the re-permeabilization of relatively small vessels (<50 µm in diameter) in a mouse model of embolic occlusion [9]. We further describe the process of pulmonary artery angiophagy according to three different stages: (1) the envelopment of the embolic specimen by the endothelial cells, (2) the translocation of an embolic specimen throughout the media, and (3) the transition of an embolic specimen to the perivascular space. These descriptions allowed us to perform a quantitative analysis of pulmonary artery angiophagy in our animal model.

In animals, the lesions of pulmonary artery angiophagy were observed in pulmonary arteries which diameters were between 17 and 4630 μm. However, the quantitative and qualitative aspects of the angiophagy were different between more proximal and more distal pulmonary arteries. The three stages of pulmonary angiophagy were observed in pulmonary arteries between 36 and 300 μm in inner diameter. In larger pulmonary arteries, we did not observe Stage III angiophagy; Stage II was observed for up to 1061 μm, and Stage I, up to 4630 μm. This suggests that the efficacy of angiophagy may decrease with an increase in pulmonary artery diameter that induces more proximal than distal chronic pulmonary artery remodeling. In very distal pulmonary arteries (<36 μm), the fact that we did not observe Stage III angiophagy may signify that the embolic specimen could have been totally removed from the adventitia into alveoli, as previously reported in mice [9], or into the lymphatic system. This further suggests that the efficacy of angiophagy may increase in more distal pulmonary arteries. However, the difference in time to Stage III angiophagy completion may also be due to larger embolic specimens in proximal arteries and smaller embolic specimens in distal arteries. Our results do not preclude the observation of more advanced stages of angiophagy in larger arteries if observed after a longer period after the pulmonary embolism. Here, our observation corresponds to pulmonary embolisms after 6 to 10 weeks of evolution.

**Figure 12:**
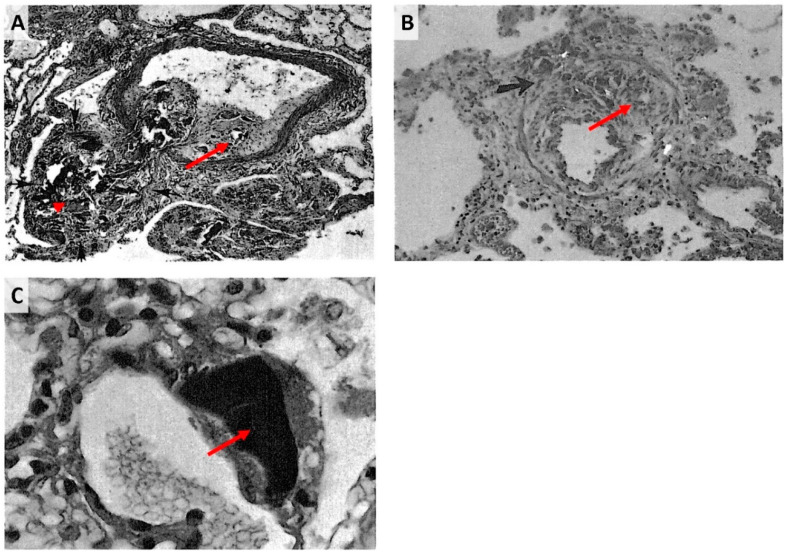
The pulmonary vascular lesions with intravenous drug abuse analyzed through the prism of angiophagy. (**A**) Stage I (red arrow) and III (red arrowhead) angiophagy in a branch of a muscular pulmonary artery occluded by cellulose emboli (darkly stained spicules) and a thrombus (outlined by black arrows). The tail of the thrombus protrudes into the parent artery, which also shows eccentric intimal fibrosis. Perivascular cellulose granulomas are present in the lower portion of the field. (Periodic acid-Schiff stain; × 80.) (**B**) Stage II (red arrow) angiophagy in a small muscular artery with a recanalized lumen, eccentric fibrosis and granulomatosis following organization of a thrombus induced by talc emboli. Spicules of optically active material are consistent with talc. Transmural extension of a foreign body granuloma is indicated by the black arrow. (Hematoxylin and eosin stain; × 150; partially polarized light.) (**C**) Stage III angiophagy (red arrow) with an unidentified homogeneous, amorphous, brown material adjacent to a pulmonary arteriole. (Hematoxylin and eosin stain; × G50.) Reprinted from The pulmonary vascular lesions of intravenous drug abuse, J F Tomashefski Jr, C S Hirsc, h Hum Pathol. 1980 Mar;11(2):133-45, Copyright (1980), with permission from Elsevier.

Angiophagy may explain the previously reported observation that it is difficult to achieve chronic pulmonary artery obstruction and CTEPH modeling in large animals with serial pulmonary embolism [4], particularly in a case of embolism with small particles [8]. Several authors reported a transient rise in pulmonary artery pressure after an acute pulmonary embolism followed by a decrease in pulmonary artery pressure within weeks, with ceramic beads in mongrel dogs [13] or with microspheres in pigs [14] and mongrel dogs [15]. Angiophagy may explain the difficulties of chronically increasing pulmonary artery pressure, as some authors reported that microspheres clustered into vessels [13,15]. This also occurred with our model, as the pulmonary artery pressure decreased within one week after each embolism, requiring several embolisms to achieve pulmonary hypertension despite the ligature of the contralateral pulmonary artery (Figure 1). In humans, Tomashefski et al. described pathological lesions in the lungs of illicit drug abusers that are compatible with Stage I to III pulmonary angiophagy lesions [2,3] (Figure 12). Indeed, they reported the presence of talc, cellulose and crospovidone emboli outside the pulmonary artery adventitia after intravenous injections and embolism to the pulmonary vessels. Of importance, the transvascular migration of the embolic constituent was already observed in bronchial arteries occluded by polyvinyl alcohol particles. In this setting, the embolic agents were enveloped by an inflammatory foreign body giant cell reaction, similar to our findings in animals. While a proportion of arteries showed persistent occlusion, some vessels became patent after embolic transvascular migration [16].

The histological analysis of human lung samples from patients who had undergone lung transplantation with histories of pulmonary thromboembolism displayed the angiophagy of blood clots and fragments of bone marrow fat tissue. The three stages of angiophagy were recognizable. However, Stage III was rarely found and, as in animals, was mainly in distal pulmonary arteries. This is consistent with our hypothesis that distal angiophagy could be more efficient with more fleeting embolic specimen attachments to peripheral wall tissues. Although these images, but also those from previous observations with different types of embolic materials (Figure 12), cannot unambiguously prove that angiophagy occurs in humans, they do provide preliminary evidence suggesting that this vascular clearance mechanism first described in mice may be conserved in pigs and in humans. Of importance, the human lung sample analyses displayed different features of thrombus evolution within each stage of angiophagy from fresh to more cellularized thrombi. We may interpret the persistent cellularization of the thrombi as an abnormal evolution of the thrombus resolution potentially leading to chronic vascular remodeling. This may be a hypothesis regarding the pathophysiology of non-curable distal forms of CTEPH that require lung transplantation.

A role for angiophagy in persistent proximal vascular remodeling should be further investigated since abnormal angiophagy could lead to chronic pulmonary artery remodeling by persistent thrombi as observed in chronic thromboembolic pulmonary hypertension [17].

We acknowledge that the description of the lesions is static and does not provide a time frame for the angiophagy process and revascularization, apart from the variation of pulmonary artery pressure oscillation after embolization in piglets, and results obtained after embolizations with non-physiologic embolic specimens should be treated with caution. The cellular and molecular mechanisms of this very recently described way of vascular clearance are still enigmatic. Future studies are required to elucidate the underlying bases of angiophagy and its kinetics. Potential triggers include the hypoxia that occurs downstream of the occlusion site, but also within the endothelium immediately adjacent to the embolus, and/or direct mechanical pressure on the endothelium by the occluding embolus or reduced shear stress attributable to the restricted blood flow [6]. At the cellular level, the term angiophagy has also been used to describe the phagocyte-like activity of endothelial cells [18]. Endothelial phagocytosis has long been known to physiologists as a function shared by endothelial cells with professional phagocytes [19,20]. Indeed, the reticulo-endothelial system [19], which also comprises the tissue macrophages and liver Kupffer cells [21], is known to engulf other particles such as apoptotic cells [22] and bacteria [23]. This phagocyte-like activity is indicated by the presence of characteristic surface-located phagocytic cups and of actin-rich phagosomes around microbeads [24]. This process may be ligand-dependent (e.g., lactadherin) [22], occurring by a mechanism called opsonization [25]. However, non-opsonized particles can also be actively taken up in vitro by endothelial cells [26]. Recently, endothelial phagocytosis was found to be largely, albeit not totally, ligand-independent and specifically controlled by the actin-nucleating formins FHOD1 and FMNL3 [23]. These mechanisms of endothelial micrometric particle engulfment may provide some insight into the vascular-scale angiophagy process. However, much remains to be investigated, but it is a matter of putting in place a foundation stone, to launch research, debate and controversies in the closed field of physiological embolus removal and the role of its dysfunction in pulmonary vascular diseases.

## 5. Conclusions

We provided the first evidence that pulmonary angiophagy may exist in human lungs as well as in piglets and in muscularized pulmonary arteries. Defective angiophagy could participate in the pathophysiology of CTEPH. In the future, it might be possible to find drugs that accelerate the angiophagy process as potential therapeutic agents for pulmonary vascular recanalization.

## Supplementary Materials

Supplementary Materials can be found at https://www.mdpi.com/2227-9059/8/11/493/s1.
