# Supplementary material for: Description, Staging and Quantification of Pulmonary Artery Angiophagy in a Large Animal Model of Chronic Thromboembolic Pulmonary Hypertension
Source: Biomedicines. 2020 Nov 11;8(11):493. doi: 10.3390/biomedicines8110493 (PMC7696066; doi:10.3390/biomedicines8110493)
Supplement: Supplementary file 1 [file biomedicines-08-00493-s001.pdf]

Supplementary Data

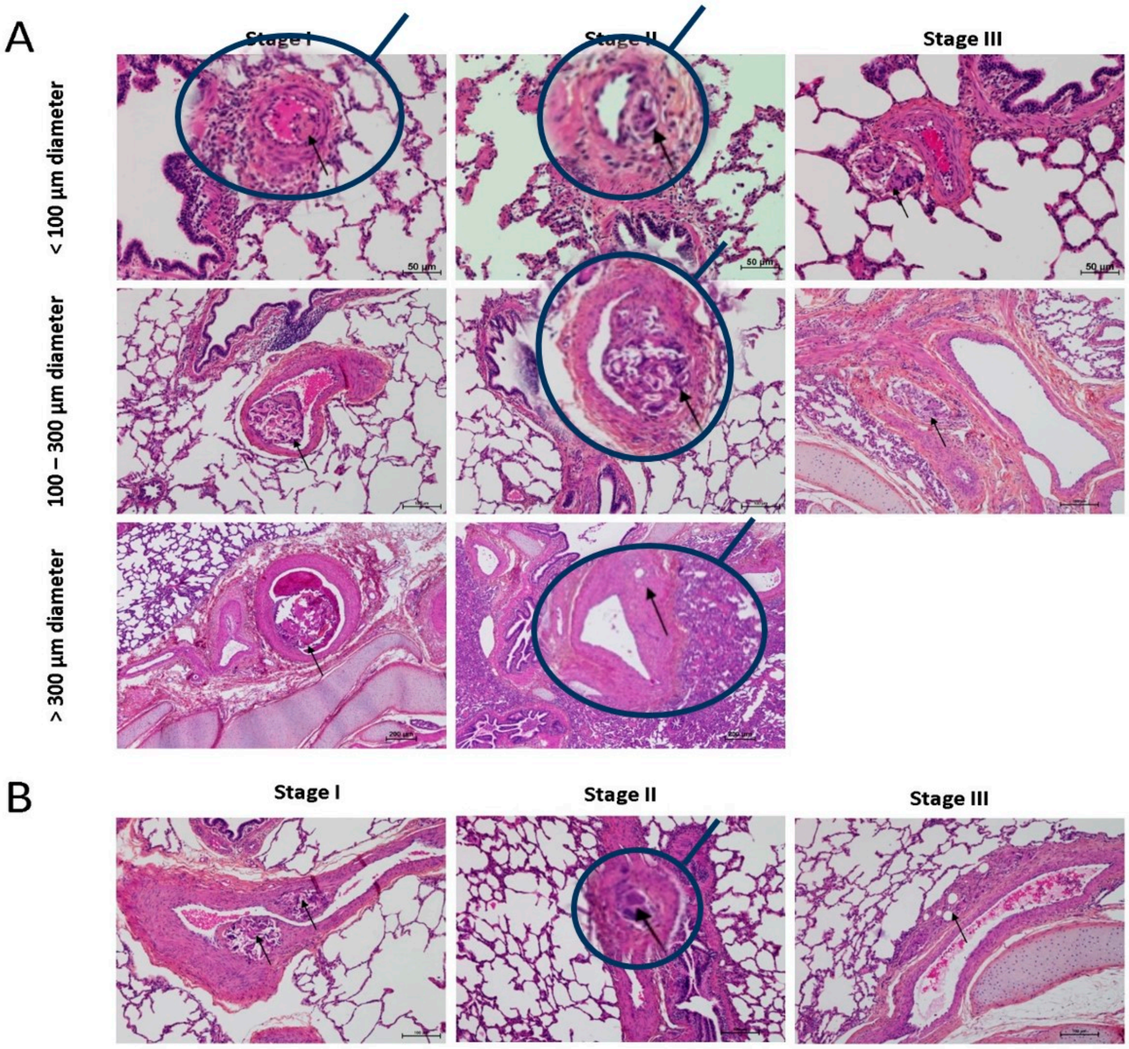

Figure S1: Histopathology of right lower lobes of the animal model of CTEPH showing axial (**A**) and longitudinal (**B**) arterial cuts of the 3 stages of angiophagy (3 rows). Arrows and zoom show embolic specimen position. Stain is hematein–eosin–saffron.

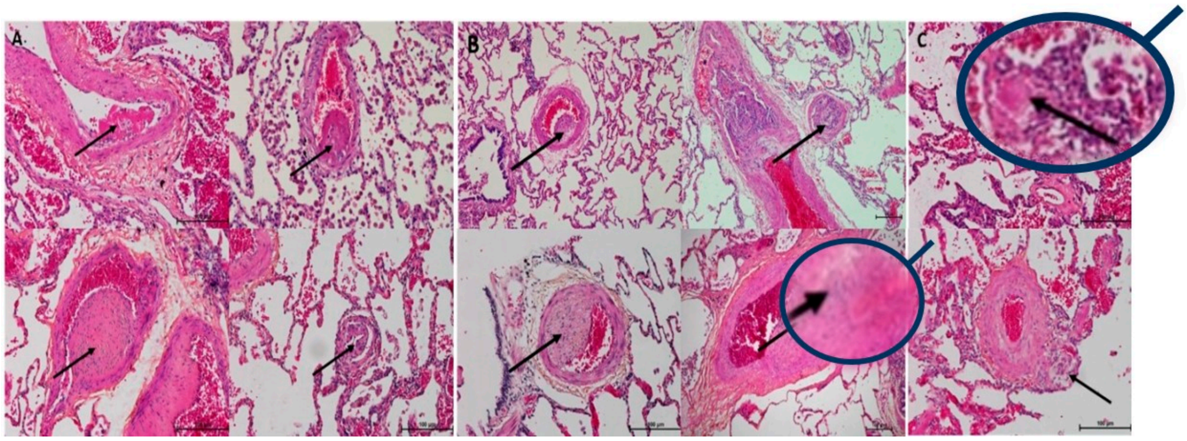

Figure S2: Histopathology of different patterns of thrombi for the 3 stages of angiophagy in patients with CTEPH, including zoom on 2 embolic specimen zones. **A** (Stage I): In the left upper panel, a recent thrombus of fibrin is poorly cellularized; in the right upper panel, the thrombus seems adherent to the media and is surrounded by endothelial cells with few cells inside the thrombus; in the left lower panel, the thrombus is, importantly, cellularized with a low rate of fresh fibrin, which reflects an aspect of fibrinous evolution; in the right lower panel, the thrombus seems entirely cellularized without fibrin or fibrosis. **B** (Stage II): The different patterns of thrombi were cellularized thrombi without fibrosis (left and right upper panels) and cellularized thrombi with fibrosis (left and right lower panels). **C** (Stage III). The thrombus is located outside the pulmonary artery (arrow) and is poorly cellularized (upper panel); the thrombus is located outside the pulmonary artery (arrow) and is cellularized (lower panel). Hematein–eosin–saffron staining.
